# Effect of three-dimensional collagen membrane culture and presence of cumulus cells on maturation of bovine oocytes

**DOI:** 10.5935/1518-0557.20230017

**Published:** 2023

**Authors:** José Pires Ribeiro Junior, Carina Diniz Rocha, João Vitor Gomes Pires, Kele Amaral Alves, Gisele Vissoci Marquini, Luiz Ricardo Goulart

**Affiliations:** 1 Nanobiotechnology Laboratory, Institute of Biotechnology, Federal University of Uberlândia, Minas Gerais, Brazil; 2 Nucleus of the Health Assistance to the Worker from Federal University of Uberlândia, Minas Gerais, Brazil; 3 Vita Human Reproduction Clinic. Uberlândia, Minas Gerais, Brazil; 4 Laboratory of Animal Reproduction, Faculty of Veterinary Medicine, Federal University of Uberlândia, Minas Gerais, Brazil; 5 Human Reproduction Sector from Federal University of Uberlândia, Minas Gerais, Brazil

**Keywords:** cumulus cells, *in vitro* oocyte maturation techniques, meiosis, 3D system

## Abstract

**Objective:**

The use of animals as experimental models has been proposed to improve the techniques applied in human reproduction clinics. This prospective and observational study evaluates the effects of the use of cumulus cells and collagen membrane on the maturation process of bovine oocytes.

**Methods:**

Design and Setting: Bovine oocytes with or without cumulus cells were cultured in maturation medium for 24 hours in the conventional system (2D), central well plates and in the three-dimensional (3D) system. Intervention: The oocytes were positioned in the collagen membrane and matured for the same period. The morphological evaluation was carried out with the parameters of maturation. Main Outcome Measure: Presence or absence of the first polar corpuscle, which were observed and classified as germinal vesicle (GV), meiosis I (MI) and meiosis II (MII).

**Results:**

The percentage of oocytes in GV was higher (*p*<0.05) in treatments without cumulus cells than those with cells. The rates of MII were higher (*p*<0.05) in the treatments with cumulus cells, independent of the culture system. In general, oocytes with presence of cumulus cells have approximately 1.7 times more chances (*p*<0.001) of reaching MII after MIV than those matured without cells.

**Conclusions:**

The presence of the cells in the cumulus is essential for the maturation process of bovine oocytes; the three-dimensional collagen membrane culture system is favorable for the maturation process of bovine oocytes.

## INTRODUCTION

The *in vitro* production (IVP) of embryos is of great scientific importance to meet the needs of women facing infertility and could be associated with increased reproductive efficiency of farm animals. However, research using human material faces difficulties due to the scarcity of material and ethical barriers. Thus, the use of animals as experimental models (Araujo *et al.,* 2014; Baerwald, 2018) has been proposed to improve the techniques applied in human reproduction clinics. In this context, the bovine species stands out for presenting similarities with antral and preantral folliculogenesis of women, and the double aptitude to take advantage of the genetic potential of the species in the development of livestock (Beletti, 2013).

The IVP techniques comprise three stages: maturation, fertilization and *in vitro* cultivation, which must take place synchronously for the IVP of embryos process to have good results. The evolution from the immature stage of a primary oocyte to the stage of complete maturation proper to undergo fertilization and sustain the initial embryonic development is called oocyte competence (Sirard, 2001; Mingoti *et al.*, 2002). The competence is only achieved through the action of cumulus cells, responsible for the production of amino acids, metabolites and growth factors that are transported to the oocyte through communicating junctions (Gilchrist *et al*., 2008), and the action of Follicle-Stimulating Hormone (FSH) that acts on the cumulus cells and stimulates the proliferation of these cells, in addition to the synthesis of steroids and expression of receptors for Luteinizing Hormone (LH) (Martins *et al*., 2008).

Nuclear and cytoplasmic oocyte maturation is one of the most important and limiting stages of the process, since it comprises the events for the oocyte to express its maximum development after fertilization (Crocomo *et al*., 2013). Among these events are cascades of activation and inhibition of enzymes, hormones and growth factors (Castro *et al*., 2011).

Usually, IVP maturation occurs in culture plates with deposition of groups of cumulus oocytes complexes (COCs) in micro drops of maturation medium, in which the COCs are in contact with the base of the plate. This traditional culture system, also called two-dimensional (2D), may present disadvantages, because the oocytes may adhere to the plate surface and impair the development of this region, preventing its contact with the nutrients of the medium, besides modifying the natural morphology of the oocyte structure, which is three-dimensional (3D).

Only 25 to 40% of oocytes submitted to the maturation process (MIV) are competent for development to the blastocyst stage, which becomes an obstacle for *in vitro* embryo production (Farin *et al*., 2007). Among the factors that influence this stage we can mention the high oxygen tension associated with light interference, temperature and absence of antioxidants naturally present in the follicular fluid (Wang & Lessman, 2002). Moreover, although the IVP technique is consolidated, the quality of embryos produced *in vitro* is still lower than those produced *in vivo*, with production rates of the usual protocols not exceeding 40%. Therefore, it is important to seek more and more tools to increase the results, so that the embryos produced *in vitro* have the potential for survival close to those produced *in vivo* (Gonçalves & Viana, 2019).

An alternative to traditional cultivation, are 3D cultivation systems, which are increasingly being tested and applied in cell culture as an alternative to increasing the development of *in vitro* culture structures. A 3D cultivation system consisting of collagen membrane that mimics the extracellular matrix environment has been used with positive results in cell associations and in maintaining cell viability (Krause *et al*., 2008). Thus, this study aims to evaluate the effects of the use of collagen membrane in the *in vitro* maturation process of bovine oocytes.

## MATERIAL AND METHODS

### Ovaries collection

The project was submitted and analyzed by the Committee on Ethics in the Use of Animals (protocol A005/18). The project was exempted from the opinion of that committee for not directly handling live animals for sample collection. The analysis is in line with federal legislation relevant to the scientific use of animals, published in Federal Ordinance No. 665/17, with attached documentation.

Ovaries of mestizo females (Bos taurus × Bos indicus), aged between 24 and 48 months, were collected at a local slaughterhouse. Then, the ovaries were transported to the laboratory in a Thermos bottle (temperature between 35-37°C) in a maximum period of 3 hours.

### Follicular aspiration to obtain the cumulus-oocyte complexes (CCOs)

In the laboratory, the ovaries were kept in a solution containing saline solution enriched with 1% fetal bovine serum and antibiotic (amikacin) at 38°C. Later, the CCOs were obtained from ovarian follicles (3 to 8 mm in diameter) by puncture with the aid of a needle coupled with a 10 mL syringe. The follicular fluid obtained was deposited in 15 mL conical tubes (Oosafe^®^ SparMED, Denmark), left to rest in a water bath (38°C for 10 to 20 minutes) until sedimentation and then transferred to Petri dishes (100 × 20 mm; Oosafe^®^) and evaluated under stereoscopic (15×; Nikon^®^, SMZ-800, Japan) for tracing of CCOs.

### Experimental design

The selected CCOs had a standardized cytoplasmic morphology (Grade I and II) according to the coverage of cells in the cumulus (with or without cells in the cumulus). Then the oocytes were washed in TCM-199 Hepes medium supplemented with bovine fetal serum (10.0%), pyruvate (0.11 mg/mL) and amikacin (83.0 mg/mL) and distributed in the following treatments: CC-2D, oocytes (n=144) with cumulus submitted to conventional *in vitro* maturation (IVM) (two-dimensional system - 2D); CC-3D, oocytes (n=126) with cumulus submitted to IVM with collagen membrane (three-dimensional system - 3D); SC-2D, oocytes (n=146) without cumulus submitted to conventional MIV (2D); and SC-3D, oocytes (n=138) without cumulus submitted to MIV in medium with collagen membrane. A total of 6 replicates were performed per treatment.

### *In vitro* Maturation (IVM)

In the conventional system (2D), the oocytes were matured in groups using central well plates with approximately 1000 µL of maturation medium. In the three-dimensional (3D) system, the oocytes were positioned on the collagen membrane and matured in drops of 1000µL of the same medium. The medium used in both the 2D and 3D systems was TCM199 supplemented. The IVM was performed in an incubator (Thermo Scientific Forma®, Series II 3110, USA) for 24 hours with 5% CO_2_ in saturated humidity at 38.5°C, according to the equipment manufacturer and standardized laboratory ambient temperature. The standardized culture time was 24 hours for all groups to analyze the maturation parameters sufficient to meet the objectives of the study, as recommended by the references used. In addition, 24 hours was standardized because it is the standard maturation time of both bovine and human oocytes, sufficient to respond to the objectives of the study.

The 3D membrane was developed in the laboratory according to cited references from the sclera of pigs. The development process was based on molecular biology recommendations for sufficient protein and ionic preservation for a favorable culture medium for cell maturation.

The sample was not coated with oil because the bovine oocyte does not need oil coating. In addition, no oil was used so as not to confuse it with the structure of the 3D membrane, but enough collagen membrane material derived from pig sclera was placed so that the oocyte did not dehydrate and there was no ionic loss.

### Morphological analysis of meiotic recovery after MIV

The evaluation of the resumption of meiosis was performed after the removal of the cumulus cell. Briefly, the CCOs were washed in a 300 µL drop of hyaluronidase (Fertipo^®^, YYA001, Belgium) for 60 seconds. They were then pipetted successively with micropipettes (Streeper Denupet, Vitromed^®^ GmBh, Canada) of different calibers (300 µm, 150 µm, and 135 µm) until the cells were completely removed. The morphological evaluation of the resumption of meiosis was performed by a single evaluator. In this evaluation the parameters of maturation with presence or absence of the first polar corpuscle were observed and classified as germinal vesicle (VG), meiosis I (MI) and meiosis II (MII) through an inverted microscope with phase contrast (Nikon^®^ TE 2000, Japan). In addition, oocytes with rupture of the pellucid zone called extrusion were evaluated.

### Metabolomics analysis

The maturation media (D0 and D1) from all experimental groups were collected and frozen for metabolic analysis. For the extraction of metabolites 100 µL of sample with 1000 µL of methanol spectroscopic grade in eppendorf of 1.5 mL was added. This mixture was incubated for 4 hours in ultrafreezer (-80°C). In sequence centrifuged for 15 min to 13000g and the supernatant transferred to another eppendorf of 1.5mL, which was packed in vacuum concentrator for 30 minutes and lyophilized. The material was stored in ultrafreezer (-80°C) until the moment of the analyses. In mass spectrometry analyses the samples were suspended in 500 µL of methanol spectroscopic grade and filtered in a 0.22 micrometer pore tip filter.

### Statistical analysis

The sample size was determined for identification, with 95% confidence (error α=0.05), a difference, if there was, of at least 50µm, between the means of the diameter of oocytes in conventional - 2D or with collagen membrane - 3D) and the presence or absence of cells from the cumulus (with or without) on the MII rate. At least 50 oocytes in each group were estimated as sufficient, with 90% test power, predicting a 50% difference between groups for the result of the presence or absence of cells from the cumulus (with or without) on the MII rate.

The statistical analysis was performed using Sigma Plot version 11 (Systat Software Inc., USA). The chromatin configuration between treatments was evaluated by Fisher’s Chi-square or exact tests. The logistic regression analysis evaluated the association of the cumulus cells and collagen matrix (independent variables) on the presence of the first polar corpuscle and metaphase II (dependent variables). The data were presented as percentages and considered significant when *p*<0.05.

## RESULTS

A total of 554 oocytes were submitted to maturation *in vitro* and later evaluated for chromatin and extrusion configuration (Table 1). The percentage of oocytes in GV was higher (*p*<0.05) in treatments without cumulus cells (SC-2D and SC-3D) than those with cells (CC2D and CC-3D). The CC-3D treatment presented lower (*p*<0.05) percentage of oocytes in MI when compared to SC-2D treatment. MII rates were higher (*p*<0.05) in CC-2D and CC3D treatments compared to SC-2D and SC-3D treatments.

**Table 1:** Chromatin configuration and extrusion rate after *in vitro* maturation of bovine oocytes in conventional (2D) or collagen membrane associated (3D) culture systems.

^‡^Treatments	Chromatin configuration (%)			Extruded (%)
	GV	MI	MII	
CC-2D	32.6 (47/144)^A^	7.6 (11/144)^AB^	59.0 (85/144)^B^	0.7 (1/144)^A^
CC-3D	27.0 (34/126)^A^	4.0 (5/126)^A^	60.3 (76/126)^B^	8.7 (11/126)^B^
SC-2D	43.8 (64/146)^B^	10.9 (16/146)^B^	44.5 (65/146)^A^	0.6 (1/146)^A^
SC-3D	46.3 (64/138)^B^	5.7 (8/138)^AB^	47.1 (65/138)^A^	0.7 (1/138)^A^

^A,B^ Different letters in the column indicate statistical significance (*p*<0.05).

^‡^Oocytes with cumulus (CC) or without cumulus (SC) submitted to conventional MIV (2D) or with collagen matrix (3D).

GV: germinal vesicle; MI: metaphase I; MII: metaphase II.

In addition, the percentage of rupture of the pellucid zone oocytes was higher (*p*<0.05) in the CC-3D treatment compared to the others (CC-2D, SC-2D and SC-3D). Logistic regression was performed to evaluate the influence of the culture system (conventional - 2D or with collagen membrane - 3D) and the presence or absence of cells from the cumulus (with or without) on the MII rate (Table 2). In general, oocytes with presence of cumulus cells have approximately 1.7 times more chances (*p*<0.001) of reaching MII after MIV than those matured without cells.

**Table 2:** Logistic regression coefficients and odds ratio for factors associated with metaphase II after maturation *in vitro* bovine oocytes in conventional (2D) or collagen matrix associated (3D) culture systems.

Factors	Coeficient	*p*-Value	Odds ratio (IC 95%)
Cumulus cells^1^	0.561	0.001	1.75 (1.25 - 2.45)
Culture system^2^	0.079	0.643	1.08 (0.77 - 1.51)
Intercepto	-0.208		

* Dependent variable: metaphasis II (no = 0; yes = 1).

1 Cumulus cells: no = 0; yes = 1.

2 Culture system: 2D = 0; 3D = 1.

The odds ratio analysis was performed among the treatments and complemented the logistic regression results (Table 3). In summary, the culture system (2D or 3D) did not influence (*p*>0.05) MII rates.

**Table 3:** Odds ratio analysis for the type of culture system (conventional - 2D or with collagen membrane - 3D) and the presence or absence of cells from the cumulus on the oocyte rate in metaphase II after *in vitro* maturation.

^‡^ Treatment - Comparisons	Metaphasis II (%)	Odds ratio (95% I.C)	*p* - value
CC-2D CC-3D	59.0 (85/144) 60.3 (76/126)	0.9 (0.5 - 1.5)	0.9274
CC-2D SC-2D	59.0 (85/144) 44.5 (65/146)	1.8 (1.1 - 2.8)	0.0186
CC-2D SC-3D	59.0 (85/144) 47.1 (65/138)	1.6 (1.0 - 2.5)	0.0592
CC-3D SC-2D	60.3 (76/126) 44.5 (65/146)	1.8 (1.1 - 3.0)	0.0132
CC-3D SC-3D	60.3 (76/126) 47.1 (65/138)	1.7 (1.0 - 2.7)	0.0427

‡ Oocytes with cumulus (CC) or without cumulus (SC) submitted to conventional MIV (2D) or with collagen matrix (3D). I.C: 95% confidence interval.

**Figure 1:**
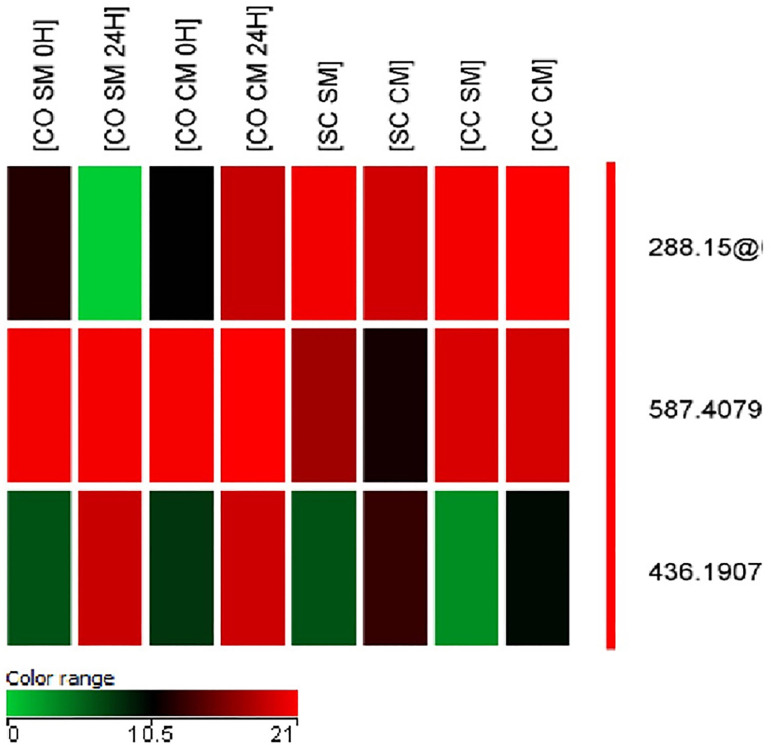
Heatmap with main metabolites identified after in vitro maturation of bovine oocytes in conventional (2D) or collagen matrix (3D) cultures in the presence (CC) or absence (SC) of cumulus cells.

^‡^Medium collected after 24 hours of culture in the absence (Control-2D) or presence (Control-3D) of collagen matrix.

After metabolomic analysis of culture media with and without collagen membrane, a total of 66 components were identified. However, only three different components between treatments (Figure 1).

## DISCUSSION

The primary result according to the questioned hypothesis refers to the possibility of offering a 3D culture medium as a promising medium for oocyte maturation in order to optimize the time and success of the procedure in human reproduction.

The present study evaluated the effect of the absence of cells from the cumulus and the adoption of the collagen membrane (three-dimensional system) in the maturation process of bovine oocytes. One of the challenges of reproductive medicine and biology is to understand the nature of molecular and cellular processes that control oocyte competence for development. This competence, acquired through maturation, is a complex process that comprises nuclear and cytoplasmic maturation and involves a deep interaction between oocyte and cumulus oophorus cells.

Cumulus oophorus cells are keys to paracrine and endocrine signaling and maturation (Bhadarka *et al.,* 2017). Variations *in vitro* maturation systems have been tested in order to preserve these connections and privilege the contact of the oocyte with the substrate. Among the variations we can mention the use of matrices and membranes that enable the maintenance of the original cell structure, unlike the flattening observed in traditional (two-dimensional) cultivation systems.

The absence of cells from the cumulus elevated the proportion of oocytes in the germinal vesicle stage. On the other hand, the presence of cells from the cumulus has benefited oocyte maturation in both culture systems (2D and 3D). The follicular microenvironment of the ovary and the maternal signs, mediated mainly by the cumulus cells, are responsible for supporting oocyte growth, its development and the gradual acquisition of developmental skills (Sutton *et al*., 2003).

Cumulus cells play a critical role during meiotic maturation *in vitro*. Previous studies have shown that naked human oocytes exhibit accelerated resurgence of meiosis *in vitro*, a deficiency in cytoplasmic ability to maintain metaphase characteristics while meiosis progresses, a propensity to activate spontaneously after interruption of meiosis, and a lack of coordination between nuclear and cytoplasmic maturation (Combelles *et al*., 2002).

In addition, the cleavage rate was reduced in a study with naked bovine oocytes (Sirard *et al*., 2006). The absence of cells from the cumulus during IVM also affects lipid metabolism of oocytes, leading to sub-optimal cytoplasmic maturation and consequently reducing their competence for development (Auclair *et al*., 2013).

In this study, the regression and odds ratio analyses proved the beneficial action of the cells in the cumulus during the oocyte maturation process. The co-cultivation with monolayer of cells from the cumulus partially restored the development potential of naked oocytes, and favored the competence of oocytes that had only the irradiated coronary (Dumesic *et al*., 2015). Many centers of human assisted reproduction have adopted oocyte denudation for the purpose of facilitating the action of promoting factors in the maturation medium. In fact, this procedure often results in oocyte impairment (Uhde *et al*., 2018).

The cells in the cumulus have promising capabilities to influence the development of oocytes by releasing secretory factors with properties of attraction to chemotaxis, prolonging the survival of oocytes and accelerating the maturation of oocytes by gene activation, in addition to improving rates of implantation and pregnancy in intra-cytoplasmic sperm injection (ICSI) (Zhu *et al*., 2015; Meseguer *et al*., 2018; Guo *et al*., 2018).

Oocytes were positively affected by maturation in the three-dimensional system due to the maintenance of their original shape. The difference between *in vitro* maturation and the *in vivo* environment may arise from the extent of communication between somatic cells and oocytes. Several studies have reported that during the pre-maturation and maturation processes there is a loss of association between oocytes and cumulus cells (Nogueira *et al*., 2007; Vanhoutte *et al*., 2008). Oocyte cumulus complexes adhere to the Petri dish in two-dimensional (2D) culture and somatic cells spread and migrate away from the oocyte. In fact, this effect alters the three-dimensional (3D) structure of COCs, thus interrupting cell-cell interactions.

Providing an appropriate environment for COC maturation in three dimensions is not easy. The density and mechanical properties of the matrix can influence *in vitro* cellular behavior (Cukierman *et al*., 2002; West *et al*., 2007). Studies using oocytes from mice and humans with 3D culture in the pre-maturation stage obtained higher cleavage rates than in two-dimensional culture in both species (Vanhoutte *et al*., 2009).

Additionally, naked oocytes from mice co-cultivated with cumulus cells in three-dimensional system showed cleavage rates and blastocysts similar to COCs cultivated in microdrops (Ma *et al*., 2007). The three-dimensional system by magnetic levitation was used in the cultivation of bovine pre-antral follicles and favored the included oocytes to complete meiosis (Antonino *et al*., 2019).

The use of collagen membranes in three-dimensional cultivation can mimic the ovarian extra cellular matrix since this structure provides mechanical support and regulates several cellular activities (Venugopal *et al*., 2006; Simian & Bissell, 2017), and benefits oocyte maturation. The first study using membranes in cell culture reported results of prolonged cell viability and maintenance of cell functional characteristics (Michalopoulos & Pitot, 1975).

Three-dimensional cultures in basement membrane gels have been successfully used for over 20 years with a variety of cell types and explant organs. The usefulness of this culture system is based on how cells and explants respond (Benton *et al*., 2009).

### Strengths and limitations

One of the strengths of the study consists of a prospective, experimental evaluation without the need for involvement with live animals or patients, that is, technically plausible and ethically correct.

Besides that, according to this study the presence of cells from the cumulus has benefited oocyte maturation in both culture systems (2D and 3D). The applicability of the success of this study consists of approving the use of cumulus cells in oocyte cultivation, in addition to offering optimized options for both 2D and 3D culture media. These results deserve to be relevant as a tool for further studies with more robust samples.

The use of 3D culture media for oocyte maturation could be a promising, effective strategy, especially to be applied in reproduction care centers. The 3D membrane was developed in the laboratory according to cited references from the sclera of pigs. The development process was based on molecular biology recommendations for sufficient protein and ionic preservation for a favorable culture medium for cell maturation. The hypothesis of using a 3D membrane was due to the 3D membrane’s property of inducing stem cells. The biological plausibility would be that this membrane with potential as a medium for transforming stem cells into tissue would induce greater oocyte maturation.

However, the authors highlight the need of future studies to assess a more robust sample to evaluate superiority or not of 3D culture for oocyte maturation and the effectiveness of extrapolating to the human species. In addition, although the sample was submitted to metabolomic analysis, with the partial identification of three components, complementary studies are being carried out to better evaluate and discuss the possible effects of these metabolites on the final outcome of oocyte maturation. The authors believe that the metabolomics analysis can bring more answers regarding the superiority or not of the 3D membrane in future studies.

## CONCLUSION

Thus, the results of this study allow us to conclude that the presence of the cells in the cumulus is essential for the maturation process of bovine oocytes. The use of the 3D collagen membrane can have a promising value in future projects to facilitate the maturation process of bovine oocytes. As it did not present inferiority in relation to the standardized culture, it may be a means, which with other projects in more robust samples, presents itself as a favorable option for oocyte maturation. Further studies are needed to enable the use of membranes in the *in vitro* production process of bovine embryos.

## ABBREVIATIONS

COCs: Cumulus-oocytes complexes

FSH: Follicle-stimulating hormone

GV: Germinal vesicle

LH: Luteinizing hormone

MI: Meiosis I

MII: Meiosis II

MIV: Maturation process

ICSI: Intra-cytoplasmic sperm injection

IVM: *In vitro* maturation

IVP: *In vitro* production

2D: Two-dimensional

3D: Three-dimensional
